# A Rare Complication of Henoch-Schönlein Purpura/IgA Vasculitis in an Adult Woman After COVID-19 Infection

**DOI:** 10.7759/cureus.42063

**Published:** 2023-07-18

**Authors:** Man-Kuang Chang, Leonardo Chang, Hsiao-Yen Kuo, Carlos A Arango

**Affiliations:** 1 Infectious Diseases, Baptist Health System, Jacksonville, USA; 2 Biochemistry, University of California Los Angeles, Los Angeles, USA; 3 Internal Medicine, Veterans Affairs Clinic, Jacksonville, USA; 4 Pediatrics, University of Florida, Jacksonville, USA

**Keywords:** immunoglobulin a, henoch-schönlein purpura, iga vasculitis, covid-19, palpable purpura, gross hematuria, covid, igav, hsp

## Abstract

Severe acute respiratory syndrome coronavirus 2 (SARS-CoV-2) infection can affect multiple organs due to activation of an inflammatory response. One of the key components of this response is the activation of immunoglobulin A (IgA), thus causing endothelial injury and inflammation. Henoch-Schönlein purpura (HSP) has been rarely reported in adult patients as a complication of the coronavirus disease 2019 (COVID-19) infection. In this report, we present a case of HSP occurring one week after the diagnosis of COVID-19 in a 23-year-old woman. Her symptoms included nausea, vomiting, diffused abdominal pain, joint pain, hematuria, and palpable purpura of the lower extremities. She was treated with intravenous methylprednisolone sodium succinate, followed by oral prednisone therapy, which resulted in clinical improvement, including resolution of abdominal and joint pain as well as skin rashes, without remaining renal complication.

## Introduction

Immunoglobulin A vasculitis (IgAV), formerly called Henoch-Schönlein purpura (HSP), is a systemic, multiorgan, immune complex-mediated leukocytoclastic vasculitis (LCV), associated with a dysregulated IgA-mediated immune response to bacterial or viral antigens [1]. Severe acute respiratory syndrome coronavirus 2 (SARS-CoV-2) infection can have multiorgan involvement mediated by an inflammatory cascade. HSP is a vasculitis commonly diagnosed in children and has been rarely reported in adult patients as a complication of COVID-19 infection [2]. Awareness of this association can support early diagnosis and intervention to prevent complications. Our objective is to bring light to IgAV as a complication of COVID-19 infection in adult patients, although it is a common association in pediatric patients.

## Case presentation

A 23-year-old woman, with no significant past medical history, developed upper respiratory symptoms with a stuffy and runny nose. She tested positive for COVID-19 by means of polymerase chain reaction (PCR). She did not receive antiviral medications. Her upper respiratory symptoms resolved with supportive therapy. A week later, the patient started to complain of bilateral knee and ankle joint pain. The following week, she developed new symptoms, such as nausea, vomiting, and diffuse abdominal pain. Despite rest, fluids, and over-the-counter analgesics, her condition did not improve. Because of worsening symptoms, she presented to the emergency room for further evaluation, where she denied fever or respiratory complications. Her initial laboratory data included an elevated white blood count of 23,000 x 103/mm3, an elevated platelet count of 387,000 x 103/mm3, and a normal serum creatinine level of 0.7 mg/dl. Urinalysis reported amber color, elevated specific gravity > 1.030, protein negative, leukocyte esterase negative, nitrite negative, elevated urine WBC per high-power field (hpf) of 10/hpf, and elevated urine RBC per hpf of 58/hpf (Table 1).

**Table 1:** Laboratory data

Laboratory	Results	Normal range
White blood cell count	23,000 x10^3^/mm^3^	6.0-17.00 x10^3^/mm^3^
Platelet count	387,000 x10^3^/mm^3^	150-350 x10^3^/mm^3^
Serum creatinine	0.7 mg/dl	0.7-1.13 mg/dl
Urine specific gravity	>1.030	1.003-1.030
Urine WBC	10 hpf	0-5 hpf
Urine RBC	58 hpf	0-5 hpf

**Figure 1:**
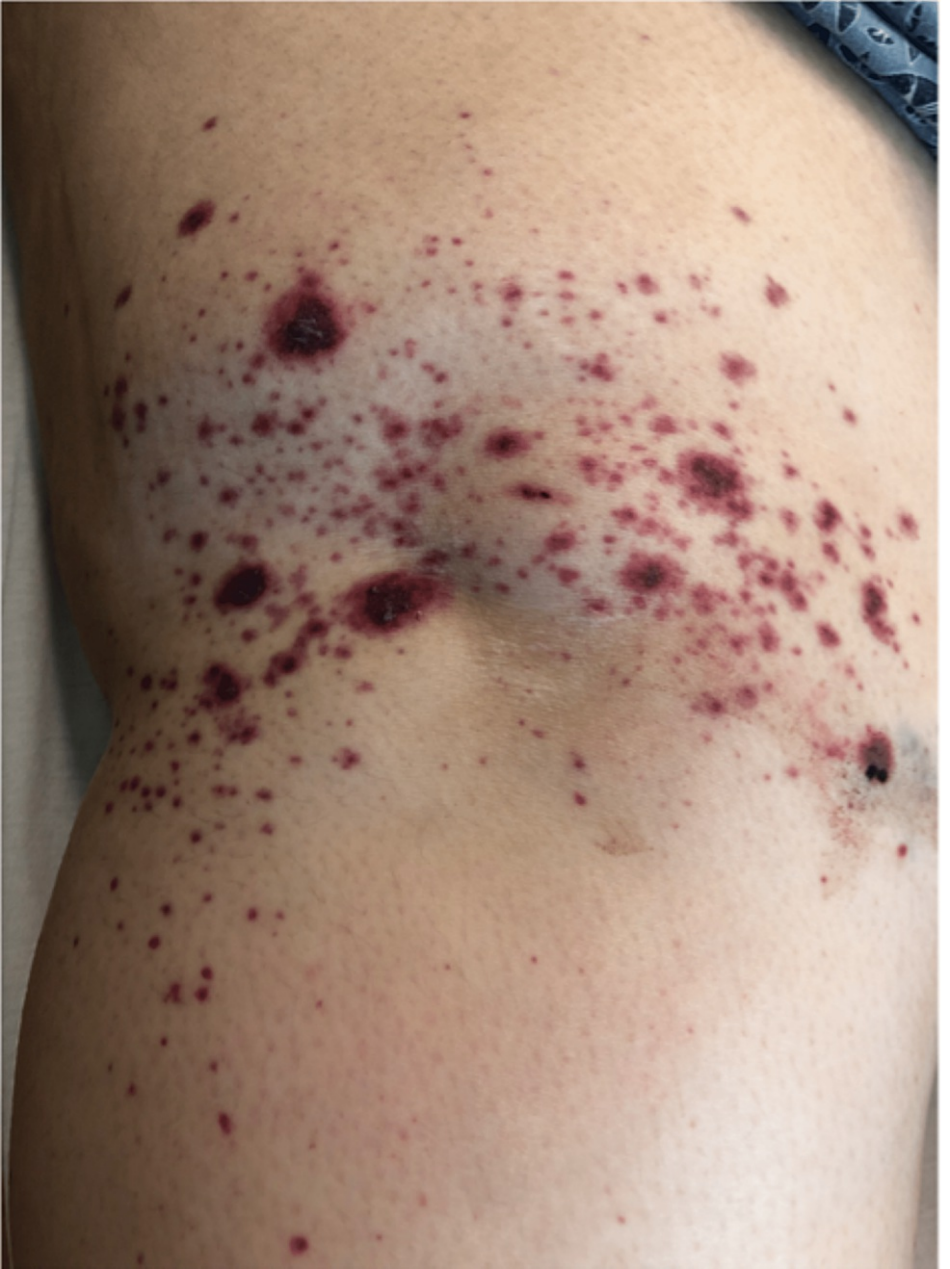
Palpable skin rash on right forearm

She developed palpable skin rashes on all four extremities during hospitalization (Figure 1).

**Figure 2:**
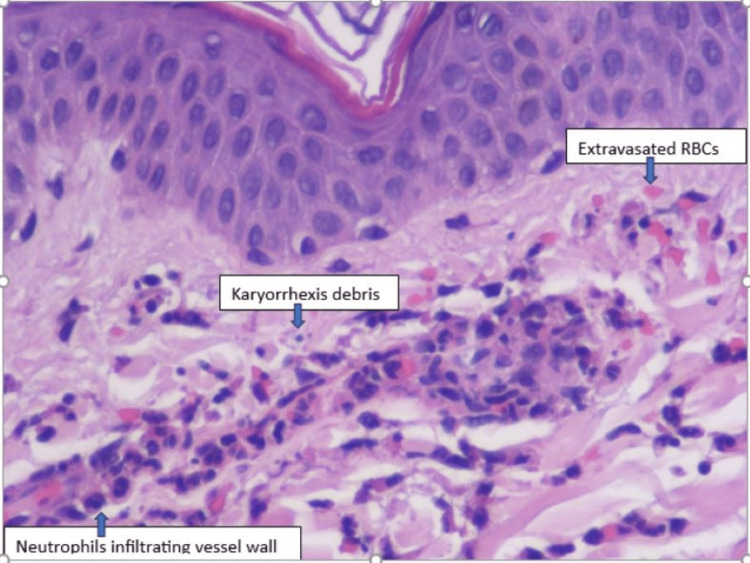
Skin biopsy of the right leg with leukocytoclastic vasculitis (LCV). Perivascular neutrophilic infiltrate (vasculitis) with occasional karyorrhexis and some extravasated red blood cells

While in the hospital, her gastrointestinal symptoms did not improve despite using intravenous antibiotic therapy with piperacillin-tazobactam for suspected bacterial gastroenteritis. She developed a purpuric rash that spread to all four extremities and back. Due to a constellation of the above symptoms, including palpable purpura, abdominal pain, multiple joint pain, and blood in urine with a history of recent upper respiratory tract infection, HSP was suspected clinically. A punch skin biopsy was obtained from her right leg. The skin biopsy showed histologic findings consistent with LCV, including endothelial swelling and perivascular infiltrates (Figure 2). A skin biopsy of the right leg showed LCV (Figure 2).

**Figure 3:**
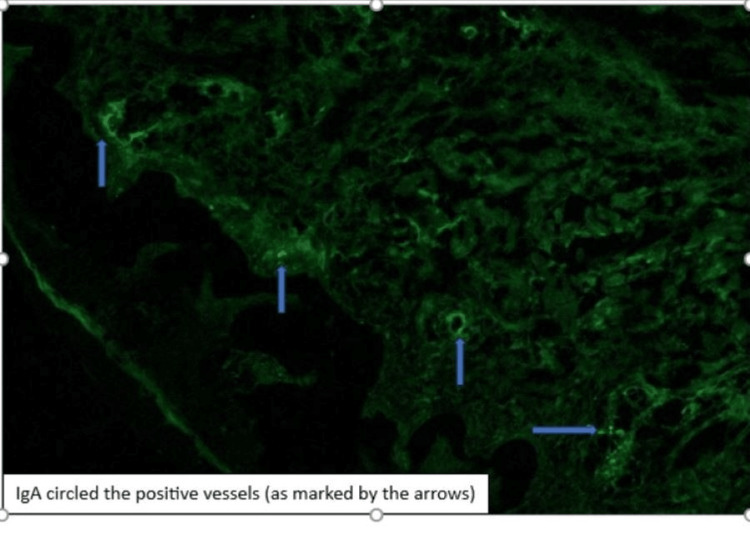
Direct immunofluorescence (DIF) studies demonstrated IgA, IgM, and fibrin deposition in small vessels

Direct immunofluorescence (DIF) studies demonstrated IgA, IgM, and fibrin deposition in small vessels, compatible with HSP (IgAV) (Figure 3).

During her hospital stay of 10 days, she was treated with intravenous methylprednisolone sodium succinate 250 mg daily, which was the standard dose for vasculitis. Her abdominal and joint pain, as well as skin rashes, improved after four days of intravenous methylprednisolone. Therefore, she was discharged home with oral prednisone 60 mg per day followed by a tapering course over two months. During the follow-up visits, her urinalysis and renal function were monitored, and they remained within normal limits. Her purpuric rash, joint pain, and abdominal pain were completely resolved after two months of a prednisone tapering course.

## Discussion

IgAV is a systemic, multiorgan, immune complex-mediated LCV that often occurs after an upper respiratory tract infection (URI) associated with a dysregulated IgA-mediated immune response to bacterial or viral antigens [1]. This IgAV presents with a constellation of symptoms, including non-thrombocytopenic palpable purpura, renal impairment, joint pain, and gastrointestinal symptoms [1]. IgAV commonly presents in children (90%) with adult cases being rare [3]. It has an incidence range of 15 children per 100,000 population vs. 1.3 adults per 100,000 population [4]. In adults, the clinical presentation includes palpable purpura (in 100% of cases), kidney disease (in 79.2% of cases), gastrointestinal (in 62.5% of cases), and arthritis symptoms (in 27.1% of cases) [4]. Serum creatinine should be obtained in adult patients with IgAV due to the associated risk of kidney disease. Almost one-third of adult patients developed kidney insufficiency within four months of clinical presentation. Kidney disease is less prevalent in children with IgAV [5].

Our patient initially presented with gastrointestinal symptoms and joint pain. She was given IV antibiotics for suspected bacterial gastroenteritis. Then, her therapy was adjusted to cover septic arthritis since she was complaining of joint pain. Later during her hospital stay, she developed a palpable purpuric skin rash and hematuria, which supported the clinical diagnosis of IgAV. A skin biopsy was obtained, and results confirmed IgAV by histopathology and DIF studies of skin biopsy.

The pathogenesis of COVID-19-associated IgAV may be related to faulty type 2 T-helper (Th2) response to this virus and the development of IgAV. This inappropriate Th2 response results in the activation of B cells and the production of antibodies. A type 3 (Th3) hypersensitivity reaction occurs with the accumulation of antigen-antibody complexes. The deposition of antigen-antibody complexes occurs with subsequent activation of the complement cascade, ultimately resulting in LCV [6].

The standard of care of IgAV consists of symptomatic management, and specific therapy to decrease the risk of complications. The prognosis in children with HSP/IgAV is excellent; however, in less than 1% of cases, they may develop long-term complications primarily chronic kidney disease (CKD) [1]. On the other hand, the risk of developing severe chronic kidney damage in the adult population is increased in comparison to children. Proteinuria and biopsy results were found to be with equal incidence in both groups (adult vs. children); however, residual CKD occurred in 31% of adults (versus 24% of children), and adults were twice as likely to go on end-stage kidney disease (15.8% vs. 7%) [7]. Currently, there is no treatment to prevent the risk of kidney damage. Most patients recover from IgAV without treatment, with symptoms resolution occurring from weeks to months. Moderate to severe cases will benefit from steroid or immunosuppressive therapy [1,3,5]. CKD is the most serious complication with long-term effects caused by IgAV [4]. Children are less likely to develop kidney disease than adults. Women who had IgAV during childhood have a three-fold poorer outcome, such as nephritis, nephrosis, or both, than males, and they may develop hypertension and proteinuria during and after pregnancy [8].

## Conclusions

HSP or IgAV has been rarely reported in adult patients as a complication of COVID-19 infection. IgAV commonly presents in children with adult cases being rare. The typical presentation includes purpuric skin rash, abdominal pain, and arthritis, but the classic rash is not the initial presenting symptom in most of the affected patients. Clinical diagnosis of HSP can be delayed, especially if the patient is in isolation due to the SARS-CoV2 infection. A biopsy of the affected organ such as skin, kidney, or duodenum that demonstrates LCV with IgA deposition confirms the diagnosis of HSP. Most children with IgAV recover without complications; few of them develop long-term complications, primarily kidney disease. In adults, the risk of significant kidney disease is increased when compared to children; however, if no kidney symptoms develop within a month after IgAV, there is a low likelihood of a poor outcome. Women who had IgAV during childhood should be carefully monitored during childbearing years, since they may develop proteinuria, elevated blood pressure, and pre-eclampsia during pregnancy more often than usual.

Treatment of HSP is mainly supportive. Most patients recover from IgAV without specific treatment. Moderate to severe cases can benefit from steroid or immunosuppressive therapy. However, there is no treatment to prevent the risk of long-term kidney damage. Further research and clinical trials are needed to develop better treatment guidelines for HSP to prevent long-term complications.
